# Toward a comparative framework for studies of altitudinal migration

**DOI:** 10.1002/ece3.70240

**Published:** 2024-08-31

**Authors:** David Vander Pluym, Nicholas A. Mason

**Affiliations:** ^1^ Department of Biological Sciences, Museum of Natural Science Louisiana State University Baton Rouge Louisiana USA

**Keywords:** animal movement, comparative biology, elevational migration, latitudinal migration, movement ecology, vertical migration

## Abstract

The study and importance of altitudinal migration has attracted increasing interest among zoologists. Altitudinal migrants are taxonomically widespread and move across altitudinal gradients as partial or complete migrants, subjecting them to a wide array of environments and ecological interactions. Here, we present a brief synthesis of recent developments in the field and suggest future directions toward a more taxonomically inclusive comparative framework for the study of altitudinal migration. Our framework centers on a working definition of altitudinal migration that hinges on its biological relevance, which is scale‐dependent and related to fitness outcomes. We discuss linguistic nuances of altitudinal movements and provide concrete steps to compare altitudinal migration phenomena across traditionally disparate study systems. Together, our comparative framework outlines a “phenotypic space” that contextualizes the biotic and abiotic interactions encountered by altitudinal migrants from divergent lineages and biomes. We also summarize new opportunities, methods, and challenges for the ongoing study of altitudinal migration. A persistent, primary challenge is characterizing the taxonomic extent of altitudinal migration within and among species. Fortunately, a host of new methods have been developed to help researchers assess the taxonomic prevalence of altitudinal migration—each with their own advantages and disadvantages. An improved comparative framework will allow researchers that study disparate disciplines and taxonomic groups to better communicate and to test hypotheses regarding the evolutionary and ecological drivers underlying variation in altitudinal migration among populations and species.

## Graphical abstract

Here, we present a brief synthesis of recent development and suggest future directions toward a more taxonomically inclusive comparative framework for the study of altitudinal migration. Our framework centers on a working definition of altitudinal migration that hinges on its biological relevance, which is scale‐dependent and related to fitness outcomes. We discuss linguistic nuances of altitudinal movements and provide concrete steps to compare altitudinal migration phenomena across traditionally disparate study systems. Together, our comparative framework outlines a “phenotypic space” that contextualizes the biotic and abiotic interactions encountered by altitudinal migrants from divergent lineages and biomes.

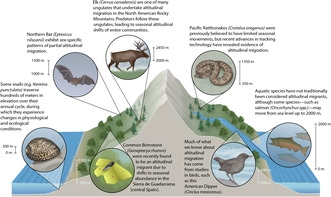

## 1 INTRODUCTION

**FIGURE 1:**
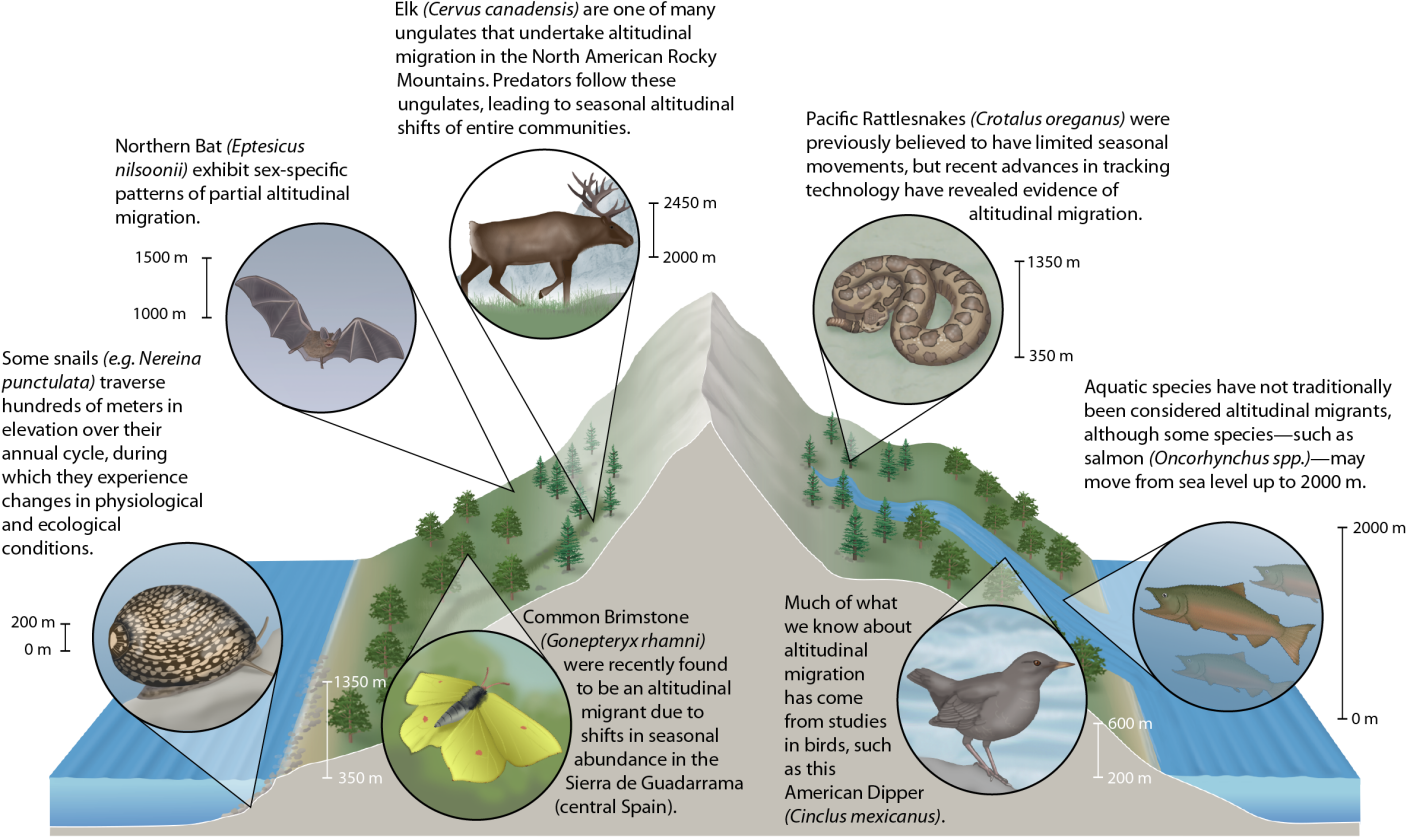
Altitudinal migration is a widespread phenomenon that occurs in many different taxonomic groups and across habitat types. Here, we show seven different examples of altitudinal migration that illustrate differences in the magnitude of altitudinal shifts as well as physiological and/or ecological changes across seasons. These examples are taken from recent studies of altitudinal migration on snails (Villeneuve et al., 2019), Common Brimstone (Gutiérrez & Wilson, 2014), Elk (Middleton et al., 2020), Northern Bat (Holzhaider & Zahn, 2001), Pacific Rattlesnake (Gomez et al., 2015), American Dipper (Morrissey et al., 2004), and Salmon (Crossin et al., 2004). Illustrations were provided by Ann Sanderson.

Since the Upper Paleolithic age >20,000 years ago, humans have been fascinated by animal movement, as evidenced by early rock art depicting animal migration (Bacon et al., 2023). From this long‐standing interest in animal movement has come a rich history in studying animal migration. Most studies of animal migration have primarily focused on latitudinal migration, although altitudinal migration—also known as elevational or vertical migration involving the seasonal movement of populations across elevational or bathymetric gradients (Hsiung et al., 2018; John & Post, 2022; Milligan et al., 2020)—has garnered increasing attention across taxonomic groups. Altitudinal migrants often pass through multiple habitats with different environmental conditions and experience a similar or even greater breadth of ecological interactions (e.g., predation, interspecific competition, interactions with parasites, etc.) compared to strictly latitudinal migrants (Williamson & Witt, 2021). As the taxonomic representation of studies on altitudinal migration has grown, so too have inconsistencies in the language used to describe this phenomenon. Here, we propose a functional definition of altitudinal migration and provide guidelines toward a comparative framework of altitudinal migration that highlights its biological importance and prevalence across taxa (Figure 1). After establishing a definition, we propose concrete steps to enable discussion among biologists studying altitudinal migration in different systems. We end with a discussion of emerging opportunities and challenges, outstanding questions in the field, and future directions to advance its study. Widespread adoption of an improved comparative framework will enable researchers to better compare and contrast emergent patterns and identify idiosyncrasies of altitudinal migration behavior among taxa and biogeographic regions.

## 2 DEFINITIONS AND NUANCES

A shared language for discussing altitudinal migration has not been used consistently in the literature. Both the term used and the definition of the phenomenon itself are inconsistent (Barçante et al., 2017), which makes it difficult to find and compare studies across disciplines and taxonomic groups. In fact, the patterns and processes that constitute altitudinal migration and the taxa considered altitudinal migrants have differed widely among studies, with most definitions focused heavily on birds (Burgess & Mlingwa, 2000: Boyle, 2017; Hsiung et al., 2018; Williamson & Witt, 2021). The terms altitudinal, elevational, and vertical migration have been used interchangeably regardless of taxa under study, even at times within the same paper (e.g., Candino et al., 2022), while other papers may not use a specific term to identify this type of migration (e.g., Harvey & Larsen, 2020; Middleton et al., 2018). A comparative framework for studying altitudinal migrations needs to start with a standardized definition and understanding of the use of the term. Most early papers (e.g., Carriker Jr., 1910; Presnall, 1935) and many recent papers on altitudinal migration are descriptive in nature; many focus on a single taxon or a handful of taxa that undertake altitudinal movements and do not define the term (e.g., Ramanzin et al., 2007; Sohil & Sharma, 2020). That said, there are many definitions of altitudinal migration that have been established in the literature, providing a good foundation for discussion (Table 1). Here, we suggest a revised definition for altitudinal migration as a seasonal round trip between two or more non‐overlapping ranges for part or all of a population along an elevational or bathymetric gradient that results in a *biologically relevant* shift in distribution. Seasonal round‐trip shifts are common in most definitions of altitudinal migration (Table 1), but what do we mean by “biologically relevant”?

**TABLE 1:** Definitions from frequently cited sources on altitudinal migration, including the term used.

Citation	Definition	Term used
Wetmore (1927)	[Regular migration up and down mountain slopes]	Altitudinal or vertical
Dixon and Gilbert (1964)	“The altitudinal movements may be termed a migration in that they show seasonal regularity, involve a considerable percentage of the first‐year age group, and involve travel of at least a few miles magnitude. The orientation component stressed by Hinde (1952:35) is manifested in altitudinal (and temperature gradient) changes rather than in compass orientation”	Altitudinal
Mead (1983)	“Very short vertical migrations made by many species in mountainous areas when they come down the mountains to reach better conditions in winter…But such movements are easily reversed”	Vertical
Stiles (1988)	“Altitudinal migrant if it shows a pronounced decrease in abundance at one altitude at the same time that it shows a roughly corresponding increase at a different altitude”	Altitudinal
Loiselle and Blake (1991)	“…seasonal movements along elevational gradients…”	Altitudinal
Johnson and Maclean (1994)	“…Moving to lower elevations in winter…”	Altitudinal
Hayes (1995)	“Any species of bird or population of the species that regularly migrates from one altitude to another on an annual basis within a biogeographical realm”	Altitudinal
Dingle and Drake (2007)	“Occur between different terrestrial elevations”	Altitudinal
Newton (2008)	[Movement in any direction down a mountain, which can also occur across large latitudinal distances]	Altitudinal
Faaborg et al. (2010)	“…If it involves movements up and down mountains”	Altitudinal
McGuire and Boyle (2013)	“Altitudinal migration commonly refers to annual return movements of all or part of an animal population between breeding and non‐breeding areas that differ in elevation”	Altitudinal
Rappole (2013)	“Altitudinal migration usually involves movement from a higher‐elevation, seasonally occupied breeding habitat to a lower‐elevation, non‐breeding, or wintering habitat. …There are also forms of altitudinal movement that are actually short‐ or even long‐distance migration, in which members of a population breed in a highland habitat but travel long distances (>1000 km) to winter at a lower‐elevation habitat”	Altitudinal
Barçante et al. (2017)	“Here, we use the term altitudinal migration for the seasonal altitudinal movement of individuals from breeding areas to non‐breeding or wintering areas, including both short‐ or long‐distance movements”	Altitudinal
Boyle (2017)	“Altitudinal bird migration involves annual seasonal movements up and down elevational gradients … Unlike obligate, latitudinal migrations, altitudinal migrations typically (1) involve short distances, (2) are controlled facultatively, and often (3) consist of partially migratory populations”	Altitudinal
Hsiung et al. (2018)	“Altitudinal migration is a type of short‐distance migration in which animals migrate seasonally between breeding and non‐breeding grounds that differ in elevation (Rappole, 2013)”	Altitudinal
Jahn et al. (2020)	“We define it as seasonal movements between high‐or low‐elevation breeding grounds to areas at other elevations within the same or a different mountain range”	Altitudinal
Pageau et al. (2020)	“Altitudinal migration is generally described as a seasonal movement from lower elevations to higher elevations for the breeding season and a downslope movement for the nonbreeding season”	Altitudinal
Kauffman et al. (2021)	“Especially in more mountainous regions, ungulates move across comparatively short spatial scales, where seasonal ranges are composed of different habitats (e.g., forest vs. alpine) but are in close proximity”	Altitudinal
Kimura (2021)	“Migration occurs not only horizontally (i.e., latitudinally) but also vertically (i.e., along mountain slopes), but these two are not mutually exclusive; altitudinal movements are always more or less associated with horizontal movements”	Altitudinal
Taki et al. (2012)	“…Altitudinal migration (i.e., movement between high elevation summer range and low elevation winter range)”	Altitudinal
Tsai et al. (2021)	“Altitudinal migration is the seasonal and repeating movement of animals along elevations (Rappole, 2013)”	Altitudinal
Williamson and Witt (2021)	“We consider elevational migration to comprise any seasonal roundtrip journey that involves elevational change between breeding and nonbreeding areas. Elevational migration may occur in conjunction with latitudinal migration, partial migration, or other forms of migration”	Elevational
John and Post (2022)	“Short‐distance seasonal movements across strata, such as altitudinal or bathymetric migrations”	Vertical
Schunck et al. (2023)	“Altitudinal migration occurs in mountainous regions around the world, where birds and other animals move up or down slopes as they follow the seasonal variations”	Altitudinal

*Note*: All are direct quotes from the text, except for those in square brackets are paraphrased.

We believe biological relevance depends on the taxon in question and whether the vertical movement directly impacts an organism's fitness—either by ensuring successful migration and subsequent reproduction, or by harming their chances for survival. Challenges to an organism's fitness can fall under two, non‐mutually exclusive categories: seasonal elevational movements that impart (1) abiotic and/or (2) biotic pressures on migrants. Considering these challenges, their interplay, and how they may act as drivers of the evolution and ecology of altitudinal migration is central to a comparative framework.

Abiotic challenges can occur when individuals move across extreme elevational gradients (i.e., Bar‐headed Geese *Anser indicus*; Scott et al., 2015), which requires adaptations for the differences in partial oxygen pressure, UV exposure, temperature, and air or water pressure (Williamson & Witt, 2021). These differences are important as they may change over shorter vertical distances compared to latitudinal distances. Vertical physical barriers, such as waterfalls, mountains, cliffs, or dams, can also impose physiological challenges to survival by blocking or constraining migratory routes or requiring additional physical exertion to cross (Cosgrove et al., 2018). Physical barriers may present different strengths of physiological or ecological change; some barriers, such as a large dam, may be impermeable to migrants (Dugan et al., 2010), while others, such as road systems, may have varying degrees of permeability (Sawyer et al., 2013). We are only just starting to understand the differences in these semi‐permeable barriers to migratory connectivity and the physiological stresses they impose (Sawyer et al., 2020). Abiotic adaptations have been studied in species that move at the extremes of elevation, but what if any abiotic adaptations do species have that move across shorter distances? Is the capacity for adaptations to abiotic challenges limited in certain taxonomic groups due to evolutionary or biomechanical constraints on movement?

Biotic challenges may include changes in habitat, intraspecific competition, predation risk, climatic changes, trophic interactions, and/or resource availability (Alerstam & Bäckman, 2018). The distance covered by altitudinal migrants varies among taxa, but the migration event typically involves annual movement between distinct ranges that do not overlap and extend far beyond the organism's home range in either site (Teitelbaum et al., 2015). On one extreme, more vagile species may move >2000 m in elevation and thousands of kilometers in latitude, which presents clear physiological challenges for survival in dramatically different environments across their annual cycle (Williamson & Witt, 2021). In contrast, some snails undertake amphidromous migrations from saltwater to freshwater habitats that cover less than a hundred meters of elevational change (Villeneuve et al., 2019), while some terrestrial species only move a few hundred meters in elevation (i.e., some *Drosophila*; Mitsui et al., 2010; *Chelonoidis* spp. Bastille‐Rousseau et al., 2017; or Trochilidae; Tinoco et al., 2009). These may seem like trivial elevational changes for more vagile taxa, but they represent dramatic shifts in ecological conditions between two distinct sites. Thus, although physiological and ecological changes are difficult to quantify, observe directly, and are not always known in studies on altitudinal migration (i.e., Tsai et al., 2021), they are nevertheless more important than changes in elevation that meet a numerical or statistical threshold between distinct sites as part of a comparative framework. Quantitative approaches that estimate biotic or abiotic “distances” are useful for categorizing altitudinal migrants, but imposing a strictly numerical change in elevation as a definition to identify altitudinal migrants without considering the biological relevance of the migration event may erroneously include or omit altitudinal migrants and bias the inferences drawn from such studies.

Even with a revised definition grounded in biological relevance and scale, there are still many nuances and potential points of confusion when discussing and classifying altitudinal migrants. For instance, animals can move both latitudinally and vertically during seasonal movements, as exhibited by monarch butterflies (*Danaus plexippus*), which migrate over the course of multiple generations from high‐elevation mountains in the Transvolcanic Belt of central Mexico to low‐elevation sites in the eastern United States and Canada during warmer spring and summer months (Kimura, 2021). There is not widespread agreement in the literature if altitudinal migration is strictly a short‐distance migration phenomenon or if it encompasses such long‐distance migration events as well. Some comparative studies or syntheses either do not address this explicitly, or include some but not all latitudinal migrants that also move in elevation (Boyle, 2017; McGuire & Boyle, 2013; Pageau et al., 2020). Other studies state it is strictly a short‐distance phenomenon (Hsiung et al., 2018), or may encompass long‐distance latitudinal migration as well (Barçante et al., 2017; Rappole, 2013; Williamson & Witt, 2021). There is no agreed upon threshold between long and short distance for an altitudinal migrant with papers at times differing widely in their definitions (Boyle, 2017). Rather, what is considered a long or a short‐distance migration may differ depending on the taxon being considered and its biology. Because both biotic and abiotic change rapidly along elevational gradients, short‐distance altitudinal migration may allow for additional access to resources via short daily trips post‐migration compared to strictly latitudinal migration events (Semenzato et al., 2021). However, migrants that move across large latitudinal expanses while also changing elevations experience similar or greater physiological and ecological changes as short‐distance altitudinal migrants. Thus, an organism may simultaneously undergo multiple forms of migration: a migratory species may be both an altitudinal and latitudinal migrant. Studies of such species should incorporate an understanding of potential drivers and ecological consequences of both forms of migration. Altitudinal migration has usually been described as a downslope movement in the non‐breeding season (e.g., Pageau et al., 2020). However, post‐breeding upslope movement to track resources that appear at higher elevations is also well‐documented across species known to migrate (Middleton et al., 2018; Shaw, 2016; Wang et al., 2023; Wiegardt et al., 2017). Furthermore, some species may breed at low elevations, but spend their non‐breeding season at higher‐elevation sites (Williamson & Witt, 2021). Other organisms may breed at low elevations and again at high elevations (Kimura, 2021). These phenomena also constitute different forms of altitudinal migration as they undergo an annual seasonal biological shift in elevation. Even within the same genus, breeding and wintering sites may differ in elevation (i.e., *Muscisaxicola*; Chesser, 2005), further connecting these as altitudinal migrants. As such, we feel that altitudinal migration should encompass both long‐ and short‐distance migrants, as well as seasonal movements in both elevational directions. Importantly, authors should explicitly state their criteria and rationale for classifying altitudinal migration states, especially when studies draw comparisons across lineages.

The complex migratory patterns of monarch butterflies illustrate another nuance of altitudinal migration: partial altitudinal migration. Not all monarch populations are migratory (Chowdhury et al., 2021), as opposed to obligate migrants where all populations within a species are migratory. Here, we follow a broader definition of partial migration, where partial altitudinal migration (including differential migration; Dingle & Drake, 2007) not only includes distinct migratory and non‐migratory populations, but also encompasses within‐population variation in migration strategies (Terrill & Able, 1988), including taxa in which only certain ages, sexes, or other demographic subunits of a population migrate (e.g., White‐ruffed manakin *Corapipo altera*; Boyle et al., 2011). The majority of altitudinal migrants are also partial migrants (Hsiung et al., 2018); conversely, many long‐distance latitudinal migrants undertake “obligate” or “complete” migration, in which each demographic class of each population participates in seasonal movements (Newton, 2012).

Altitudinal migration is a specific form of animal movement. Importantly, compared to broader movement, altitudinal migration is seasonal and cyclic with individuals and populations moving between distinct non‐overlapping areas at different elevations, which distinguishes it from other forms of movement where populations may shift their elevational distribution, but not in concert with seasonal change across the year (i.e., dispersal and irruptions). Some cyclic daily movements may occur across vertical gradients, such as “hilltopping” in some insects, in which they form daily leks on top of hills or mountains (Kimura, 2021), and aquatic animals undergoing “diel vertical migration,” in which individuals change water depth between night and day (Chapman et al., 2012). However, these are daily rather than seasonal movements at different elevations or depths, and therefore, do not fall under the definition of altitudinal migration outlined in this manuscript.

**FIGURE 2:**
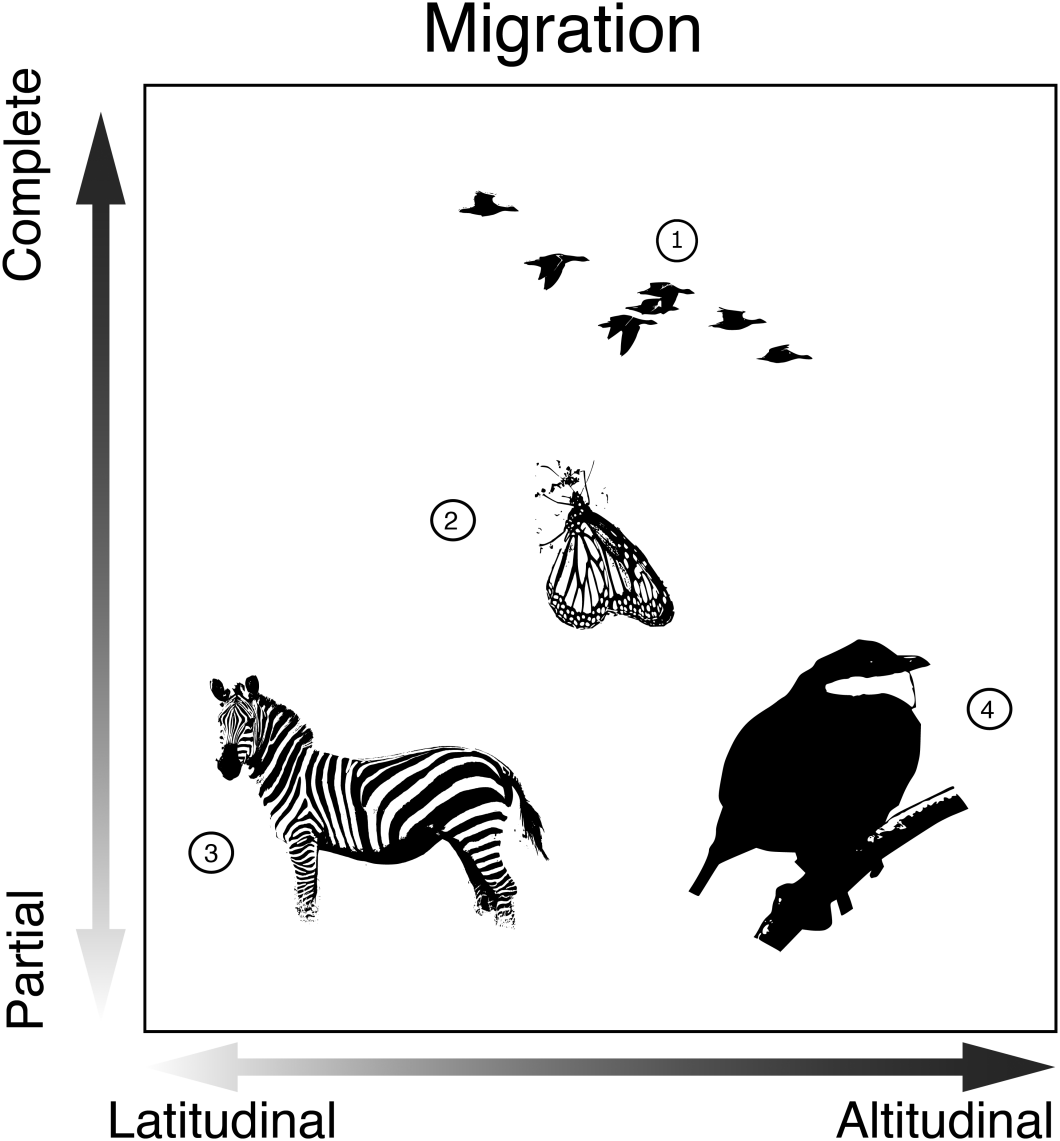
A simplified, multivariate space that conceptualizes migration behavior continua. Though many animal populations can be classified as either altitudinal or latitudinal migrants and obligate or partial migrants, many taxa and/or populations do not fit neatly into a single categorization. Rather, migrants may undertake both latitudinal and altitudinal migration, while populations or demographic classes within a species may vary in migratory behavior, such that species can be placed in a hypothetical “migration space” with continuous axes that describe variation in different aspects of migration. Determining what space an organism occupies on this simplified model is an important first step in a comparative framework. Examples include (1) Geese are typically thought of as “traditional” latitudinal migrants, moving from their high‐latitude breeding range to a low‐latitude wintering range, but may also move across large vertical distances during their migration (e.g., Bar‐headed Goose *Anser indicus*). (2) Monarchs *Danaus plexippus* show a complex partial migration pattern that transverses latitudinal and altitudinal distances, varies by population, and spans multiple generations. (3) Plains Zebra *Equus quagga* is well known as part of the great Serengeti migration that is latitudinal, but does not change in elevation. However, at the species level it is a partial migrant as some populations are resident. (4) White‐ruffed Manakin *Corapipo altera* is a partial altitudinal migrant that does not travel long longitudinal distances; only some age and sex classes migrate to lower elevations during their non‐breeding season. Photos of the White‐ruffed Manakin, and Monarch were taken by David Vander Pluym, Plains Zebra photo was provided by Joachim Huber, CC BY‐SA 2.0 https://creativecommons.org/licenses/by‐sa/2.0 via Wikimedia Commons. Geese photo was provided by Thermos – Own work, CC BY‐SA 2.5, https://commons.wikimedia.org/w/index.php?curid=1387483.

The difference between migration and movement is often straightforward in many organisms. However, migration and movement exist along a behavioral and ecological continuum, and distinguishing migration from the broader study of movement is not always straightforward, especially among short‐lived species that may undergo diapause (e.g., some flies in the family Calliphoridae; Kimura, 2021). Within an organism's lifetime they may undergo both movement and migration. For example, two polar breeding birds—Ivory Gull *Pagophila eburnea* in the Arctic, and Snow Petrel *Pagodroma nivea* in the Antarctic—both breed on high remote rocky outcrops (up to 2000 and 2500 m, respectively) and make regular foraging trips to the ocean (Carboneras et al., 2020; Mallory et al., 2020), which is a non‐migratory movement. However, the young that are born at high‐elevation nesting sites and return to breed after spending the non‐breeding season at sea level have been considered altitudinal migrants in the case of the Ivory Gull, whereas no Antarctic birds—including the Snow Petrel—are considered altitudinal migrants (Barçante et al., 2017; Hsiung et al., 2018). Nomadic species provide an extra challenge as they may require years of tracking in order to determine whether their movements are irregular rather than regular and seasonal (Teitelbaum & Mueller, 2019). Many descriptive studies suggesting a species is an altitudinal migrant have lasted under a year, which may have subsequently miscategorized single‐year nomadic movements as regular, recurring migration events (Schunck et al., 2023). The study of migration is embedded within the broader study of animal movement, but determining where the narrower study begins is sometimes difficult due to complex life histories involving seasonal or ontological changes in elevation. As part of a comparative framework, one can envision a multivariate space to describe how organisms vary in their migratory behavior. For example, it may be helpful to place organisms along continua that describe variation whether organisms exhibit latitudinal migration, altitudinal migration, or both in conjunction with whether those taxa are complete or partial migrants (Figure 2). Additionally, considering abiotic and biotic interactions can add additional dimensions by which to compare and contrast migrant taxa.

**FIGURE 3:**
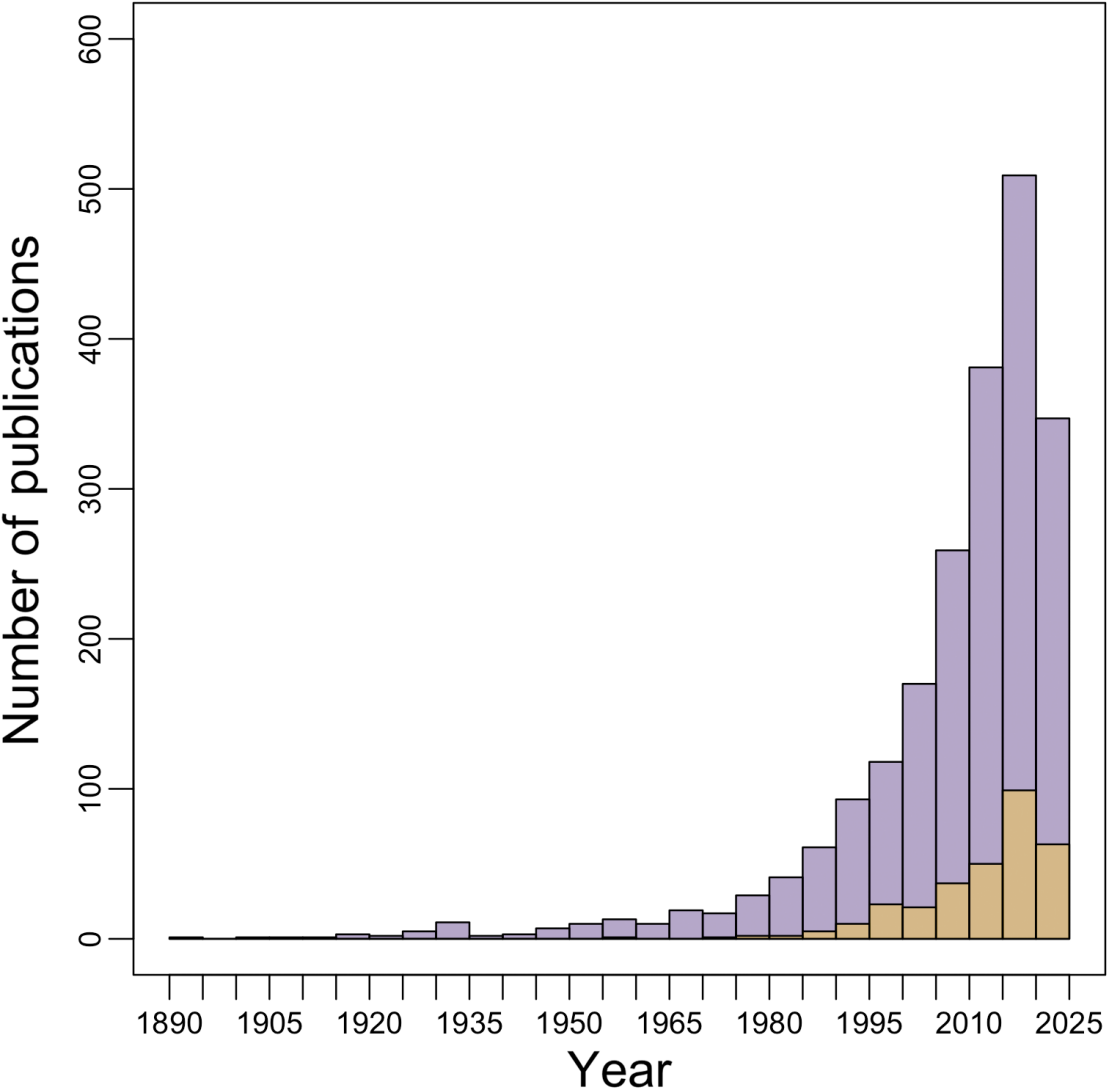
Histogram comparing the number of Google Scholar hits for search terms “altitudinal migration” in purple and “elevational migration” in yellow as quantified via the program Publish or Perish. Extraneous (e.g., those using the terms to refer to distributional shifts not related to annual cycles, which equates to ~27% of “elevational migration” hits and ~19% of “altitudinal migration hits”) and duplicate hits (~1% from each) or citations missing years have been removed. Both terms have seen a steady increase in the number of publications over time: “altitudinal migration” has an estimated growth rate of 5.51%, whereas “elevational migration” has a growth rate of 10.34%.

Finally, establishing a consistent language for the field is important to help improve the visibility and searchability of the primary literature involved. Though “vertical” or “elevational” migration may be seen as more accurate descriptors of this type of seasonal movement across biomes and taxonomic groups, the term “altitudinal migration” has historical precedence (i.e., Carriker Jr., 1910; Presnall, 1935; Todd & Carriker, 1922) and is more frequently used in the literature. We used the program Publish or Perish (Harzing, 2007) to quantify temporal trends in Google Scholar hits for publications that use the terms “altitudinal migration” and “elevational migration.” We removed any articles from this data set that did not refer to seasonal movements of organisms across altitudinal gradients. Although both terms received some extraneous and duplicate hits, we found that altitudinal migration was established as a term far earlier and has been used far more often than “elevational migration” (Figure 3). Specifically, we recovered 2228 Google Scholar search hits for “altitudinal migration” compared to 335 for “elevational migration” (searches done on August 15, 2023). Also, the term altitudinal migration is more established across reviews and papers that explicitly define this phenomenon (Table 1). Using this data set, we also calculated the log‐linear model of publication growth rate for both “altitudinal migration” and “elevational migration.” We found that “altitudinal migration” has an estimated growth rate of 5.51%, whereas “elevational migration” has a growth rate of 10.34% (see Data S1 for R Code). Growth rates for both terms are higher than what has been estimated for all sciences (4.10%; Bornmann et al., 2021) and for the life sciences (5.07%; Bornmann et al., 2021), indicating strong interest in this field of research.

Although “altitudinal migration” and “elevational migration” have often been used interchangeably, we recommend using the more commonly used term altitudinal migration. We advocate for its use because it has historical precedent, and it better encompasses terrestrial and aquatic migrations. Although altitude is commonly referred to as the height above sea level, altitude also refers to the height above the sea floor (Jardine, 1986). Thus, as a unifying term altitude is more accurate (i.e., height above sea floor or height above sea level) than elevational. “Vertical migration” is perhaps more applicable across both terrestrial and aquatic biomes, but is used predominantly in aquatic systems (over 141,000 Google Scholar search hits on August 15, 2023) to describe a different phenomenon of daily (diel) movement across multiple trophic levels up and down the water column rather than the seasonal movements we focus on here (Chapman et al., 2012).

Use of a single term not commonly used elsewhere in the literature will aid researchers and search engines in identifying information on this phenomenon rather than often‐conflated terms (Leeming, 2023). That is not to say that the term altitudinal migration does not have drawbacks. The term is frequently used to discuss changes in elevational limits, especially regarding plants that move upslope in response to climate change (Zhang et al., 2023). Upslope shifts in response to changing climate conditions is sometimes also referred to as elevational migration (Kellner et al., 2023). While this may add confusion, the use of the terms in botany differs from its use in animal migration and while time‐consuming researchers can filter these from searches for animal migration. Confusingly, within the animal ecology and evolutionary literature, the terms are also used for range shifts of animals along elevational gradients (e.g., Deng et al., 2021). This latter usage differs from seasonal migration discussed here and though it is usually apparent which usage is being applied to a given paper, nonetheless care must be given to filter these out. This issue is also more prevalent with the term elevational migration: from Figure 3, we dropped ~27% of hits for elevational migration, while only ~19% from altitudinal migration due to the hits not referring to seasonal animal migration. “Altitudinal migration” may also seem a misnomer due to the definition of altitude in aviation, yet we nonetheless recommend its use to promote a consistent vocabulary to facilitate dialogue across disciplines and taxonomic groups.

## 3 GUIDELINES TO IMPLEMENT A COMPARATIVE FRAMEWORK IN STUDIES OF ALTITUDINAL MIGRATION

Up until now, we have focused mostly on linguistic nuances underlying the study of altitudinal migration. It is important to establish a common scientific language to enable discussion among research groups that study different taxonomic groups across various parts of the world. Once a shared language is in place, we believe that there is much to be gained by comparing altitudinal migration phenomena among lineages and biomes. Here, we set forth a set of concrete steps that will help guide a comparative framework and enable comparisons between traditionally disparate focal systems. In doing so, our goal is to collectively establish an “*n*‐dimensional” phenotypic space (i.e., Figure 2) of altitudinal migration phenomena that considers similarities and differences in migratory routes, ecological interactions, and physiological challenges encountered during altitudinal migration.

1. Foremost, it is important to establish whether the movement in question is indeed “altitudinal migration” or some other form of movement. As discussed above, we suggest that altitudinal migration must involve seasonal movement that is linked to fitness outcomes between one or more sites across an annual cycle for some proportion of a set of populations. In determining whether a focal taxon is an altitudinal migrant or not, researchers must consider if the evidence is sufficient or if further studies of the annual cycle are required to confidently classify it as altitudinal migration.
2. Consider the migratory route that is traveled during the altitudinal migration event. It is important to contextualize both the vertical and horizontal profile of the path traveled in light of the movement ecology of the focal taxon. One should also consider physical or ecological barriers in the migratory route that may impede movement. This corresponds to the “x axis” in Figure 2, which describes a continuum of latitudinal and altitudinal migration.
3. Consider plasticity in altitudinal migration within and among populations. In many populations, only certain demographic classes or sexes may undertake migration in a given year. Also, within species, populations may vary in their migratory routes and propensity for altitudinal migration. This corresponds to the “y axis” in Figure 2, which describes a continuum of partial versus complete migration.
4. Consider the abiotic conditions encountered by the organism over the altitudinal migration event. This may include, but is not limited to, physiological challenges such as changes in oxygen availability, light availability, and temperature. Together, changes in abiotic conditions may impose physiological stress on the organism as they follow their migratory path. By comparing abiotic challenges among focal taxa, researchers can accentuate adaptations and evolutionary developmental constraints present among altitudinal migrants. This is not depicted in Figure 2, but represents another potential axis of phenotypic space for altitudinal migration.
5. Consider the biotic interactions encountered by the organism over the altitudinal migration event. This may include, but is not limited to, changes in diet and food availability, predator presence or absence, parasites, and competition for other resources. As biotic conditions change across elevational gradients, consider if an organism's migration ecology involves niche tracking or is a niche shift in resource use. Ecological interactions may be asymmetrical, such that altitudinal migrants may impact sedentary species that they seasonally overlap with while tracking their ecological niche. By comparing these biotic interactions, researchers will be better able to understand how altitudinal migrants contribute to ecosystem services.

## 4 TAXONOMIC PREVALENCE

Across the tree of life, from ocean depths to mountain peaks, various animals change their elevational distribution and/or depth between seasons (Figure 1; Hsiung et al., 2018; Milligan et al., 2020). Studies on altitudinal migration have largely focused on insects (Kimura, 2021), mammals (primarily ungulates and bats; McGuire & Boyle, 2013), and especially birds (Barçante et al., 2017), whereas altitudinal migration research on other terrestrial organisms (e.g., non‐avian reptiles, amphibians, and non‐insect invertebrates) is comparatively scarce (Hsiung et al., 2018). Altitudinal migration is widespread and can even bridge aquatic and terrestrial biomes in some species (e.g., salmon *Oncorhynchus* spp.). Although salmon and other diadromous taxa are not typically included in discussions of altitudinal migration, or are mentioned only in passing (e.g., Hsiung et al., 2018), they do undertake altitudinal migration as some species move from below sea‐level up to ~2000 m in elevation in the mountains of western North America over the course of their breeding cycle (Crossin et al., 2004). Further connecting the land to the ocean at a smaller scale, terrestrial crabs also make seasonal migrations from several hundred‐meter‐high mountains to the ocean (i.e., *Johngarthia lagostoma* Hartnoll et al., 2010), while other species may only move from the ocean to a nearby beach (i.e., *Coenobita brevimanus* Nio et al., 2019). Correspondingly, they experience many of the same challenges and drivers as “traditional” (i.e., terrestrial) altitudinal migrants, such as changes in air pressure, habitat type, and predation risks. Anadromous and catadromous fish as well as crabs moving up mountains also connect the study of altitudinal migration to aquatic species that may seasonally move across bathymetric gradients (Aguzzi et al., 2013; Milligan et al., 2020). Many of these bathymetric movements are also seasonal and involve a biologically relevant shift in distribution. They have been included in other discussions of vertical/altitudinal migration (as vertical migration; John & Post, 2022) and also fit in our broad definition of altitudinal migration. With respect to biomass, the largest form of vertical movement on the planet is diel vertical migration (Brierley, 2014). Many of these organisms are extremely short‐lived, and these movements take up a significant portion of their lifespans. As previously stated, however, this is still a daily rather than seasonal movement, and thus does not fit within the definition of altitudinal migration used in this manuscript. Rather, this is a distinct, well‐studied phenomenon with its own specific term.

We believe there are interesting insights to be gained from comparisons of altitudinal migrants across terrestrial and aquatic systems. For example, there are considerable differences between terrestrial and aquatic environments, especially in regard to changes in pressure and light levels. Though migrants across an elevational and a bathymetric gradient experience changes in pressure, these changes do not scale the same. On average, a change in 10 m of oceanic water depth results in an increase of 1 atmosphere, while a similar change of altitude results in a negligible pressure change; thus, an organism must respond differently between air and water pressure and raw vertical differences may not be directly comparable between biomes. The amount of light and UV exposure also differs significantly between the two realms, with light availability sharply decreasing in water (Mobley, 1994), while higher elevations see higher levels of UV radiation. Yet, multiple aquatic species traverse these realms (Hsiung et al., 2018) and have been considered under the same umbrella term (John & Post, 2022). Despite these differences, animal seasonal migration across bathymetric gradients shares many similarities with seasonal migration across elevational gradients. Both face challenges in biotic (e.g., differing habitat, escape from predation) and abiotic changes (e.g., seasonal temperature). These similarities provide common ground for discussions between aquatic and terrestrial biologists. For example, comparisons between aquatic and terrestrial migrants may reveal whether altitudinal migrants share similar evolutionary associations, such as body size differences between residents and migrants, or whether there are differences in the tempo of life history traits.

Ignoring the nuances that we outline here risks misclassifying the altitudinal migratory status of the taxa at hand. We believe that many reviews and databases concerning altitudinal migration consistently underestimate the number of species that are altitudinal migrants (Barçante et al., 2017). This underestimation is in part due to a lack of data on many species, but is also related to how altitudinal migration is defined. We suspect this is especially true when studies exclude populations that are altitudinal migrants as well as latitudinal migrants, which would constitute a type II error or a “false negative” (i.e., Bar‐headed Goose, *Anser indicus*, is included as an altitudinal migrant in some but not all reviews). However, the reverse pattern also occurs: sometimes species' irruptive movements (i.e., Pine Siskin, *Spinus pinus*; Boyle, 2017) or opportunistic movements to avoid inclement weather (i.e., those mentioned in O'Neill & Parker, 1978) are sometimes misclassified as altitudinal migration, which would be a type I error or a “false positive.” Without a consistently applied definition, studies of altitudinal migration—especially macroevolutionary studies that incorporate hundreds or even thousands of taxa—may inconsistently classify migratory behavior, which could lead to erroneous inferences and biases. Regional biases also exist: some geographic regions have received more attention than others (Boyle, 2017), both in terms of the number of publications and the rigor of scientific study (Schunck et al., 2023). A consistent definition also allows comparisons among regions that may have heretofore been using different definitions of what constitutes an altitudinal migrant.

## 5 FUTURE OPPORTUNITIES: COMPARATIVE INSIGHTS AND ADDRESSING CHALLENGES

Recent reviews have highlighted some of the persistent challenges involved in studying altitudinal migration (Barçante et al., 2017; Hsiung et al., 2018; Jahn et al., 2020; Kimura, 2021; Williamson & Witt, 2021), which recent advances highlighted here may help address. Here, we identify longstanding questions in altitudinal migration and then consider how new technologies or approaches may help answer these questions.

1. What is the full taxonomic extent of altitudinal migration? Whether or not many taxa undertake altitudinal migration remains unknown, especially in the global south (i.e., Guaraldo et al., 2022; Maicher et al., 2020; Rappole et al., 2011). Many studies and sources on altitudinal migration have lacked scientific rigor, are descriptive, or are from “gray” literature and are not easily found (McGuire & Boyle, 2013; Schunck et al., 2023), all of which hampers our knowledge of the true extent of altitudinal migration. Combining macroevolutionary analyses in a phylogenetic comparative framework with population‐level studies will reveal how altitudinal migration contributes to diversification at different taxonomic scales. A deeper understanding of the taxonomic diversity of altitudinal migration will also clarify how ecological and evolutionary drivers may overlap and contrast with latitudinal migrants (Barçante et al., 2017; Hobson et al., 2019; Hsiung et al., 2018; Pageau et al., 2020). It will also clarify if certain taxonomic groups are more likely to be altitudinal migrants, or whether certain groups are better studied for altitudinal migration (i.e., birds)?
2. How do the drivers of altitudinal migration differ among regions? Each mountain range or bathymetric feature has a unique geological history and corresponding evolutionary pressures driving the gain and loss of altitudinal migration. As such, patterns of evolution underlying variation in altitudinal migration may vary substantially among regions that vary in latitude or their surrounding biome (Rahbek et al., 2019). The distinct age and origin of each landscape feature can contribute to differences in elevational zonation, climate, and the potential for altitudinal migration to evolve. Studies of altitudinal migration have been highly concentrated in a few select biogeographic realms and countries, especially in North America (Hsiung et al., 2018; Pageau et al., 2020; Schunck et al., 2023). Janzen's (1967) hypothesis that mountain passes are “higher” in the tropics has received substantial interest (e.g., see Smith, 2018 commentary), but how this relates to altitudinal migration has received far less attention (but see Gadek et al., 2018). Applying this concept to altitudinal migration, pronounced elevational zonation of habitat in the tropics may induce altitudinal migrants to move smaller elevational distances (but similar ecological “distances”) compared to temperate species. Expanding the geographic scope of altitudinal migration studies will undoubtedly reveal novel patterns and comparisons among regions with altitudinal migrants.
3. What impacts does altitudinal migration have on speciation and diversification? Are certain ecological or morphological traits associated with evolutionary gains or losses in altitudinal migration? Various studies have considered how the evolution of different migratory states may impact speciation, both empirically at the population level (Gómez‐Bahamón et al., 2020) and through a theoretical lens (Winker, 2010). However, little is known regarding how altitudinal migration may impact rates of speciation or diversification. It is not known if ancestral populations of altitudinal migrants were latitudinal migrants or sedentary, though it is likely there have been multiple gains and losses of migratory status in many clades (Chesser, 2000). One might hypothesize that changes in altitudinal migration may lead to a reduction in gene flow—as is seen in latitudinal migration—but there are few empirical papers that have explicitly tested this hypothesis (but see Tigano & Russello, 2022; Williamson et al., 2024). Future studies could clarify how changes in altitudinal migration impact patterns of gene flow and whether there are generalizable or idiosyncratic patterns across lineages regarding whether altitudinal migration character states or transition rates are associated with speciation rates.
4. How is anthropogenic change impacting altitudinal migration and migrants? Migrants and especially altitudinal migrants experience such a wide breadth of environmental conditions, they are severely impacted by anthropogenic change at different elevations and may be more subjected to declines caused by forest fragmentation (Loiselle & Blake, 1992; Runge et al., 2014). For example, many altitudinal migrants are reliant on narrow elevational bands of habitat, which is rapidly shifting upslope as our planet warms (Chen et al., 2011; Maicher et al., 2020). Changes in the elevational distributions of ecosystems and their constituents may induce new ecological interactions, such as the introduction of avian malaria to the Hawaiian Honeycreepers, many of which are altitudinal migrants and become infected with malaria during their non‐breeding season at lower elevations (Eggert et al., 2008; Guillaumet et al., 2017). Anthropogenic change has also induced various phenological shifts, which can harm altitudinal and latitudinal migrants due to a mismatch in the timing of resource availability (Green, 2010; Inouye et al., 2000). Despite the potentially pernicious impacts of land use and climate change on altitudinal migrants, empirical studies that address this harm and our general understanding of anthropogenic impacts on altitudinal migrants are still limited in many systems (but see Powell & Bjork, 2010; Adams, 2018; John & Post, 2022). Amid a rapidly changing climate, our understanding of how differences in mobility and physiology impact the capacity for altitudinal migration among different animal groups is lacking.
5. What comparisons can we draw between altitudinal and latitudinal migration? Migration is taxonomically widespread, and a broader comparative framework for studies of altitudinal migration allows for more nuanced comparisons of animal movement (Dingle & Drake, 2007). As in latitudinal migration, subcategories of altitudinal migration can be differentiated and compared along continuous axes: altitudinal migration can be partial or complete, short‐ or long‐distance, aquatic or terrestrial, and facultative or obligate. How do these different continua interact and compare to latitudinal migrants? Are certain types of altitudinal migration more common among certain lineages or biogeographic regions? Are there shared paths of altitudinal migration (i.e., “flyways”) as there are in latitudinal migrants? Do barriers to movement impact altitudinal migrants in a similar or different way to that observed in latitudinal migrants? Do altitudinal migrants share similar navigation strategies with latitudinal migrants? Aside from salmonids, little is known about navigation cues and mechanisms in altitudinal migrants (but see Berger et al., 2022), while comparisons between strictly altitudinal migration and latitudinal migration are lacking.
6. What can we gain from comparisons of altitudinal migration patterns across taxonomic groups? Are certain ecological or morphological traits associated with evolutionary gains or losses in altitudinal migration? Across mammals and birds on a macroevolutionary scale, migrants are on average more similar to each other for several life‐history traits than they are to their more closely related resident counterparts, suggesting evolutionary constraints on migratory phenotypes (Soriano‐Redondo et al., 2020), but does this pattern hold for ectotherms or partial migrants? Comparative studies have been a fruitful area of research in latitudinal migration research (i.e., Soriano‐Redondo et al., 2020), however, this topic has received little attention for altitudinal migrants (Pageau et al., 2020). Questions such as how ectotherms address the challenges of traveling long distances across elevational gradients compared to endotherms have yet to be addressed. Though birds are comparatively well studied in terms of altitudinal migration, we have only scratched the surface of how they are physiologically adapted to the changes in partial pressure and oxygen levels (see Williamson & Witt, 2021). How other organisms manage these changes, and if they have physiological changes is largely unknown (but see Jacobsen, 2020). Comparative studies at different taxonomic scales will reveal how ecological, physiological, and morphological variation among lineages impacts how altitudinal migration has evolved in different animals.
7. What is the nature and extent of ecosystem services provided by altitudinal migrants? Animal movement across ecosystems affects food webs, nutrient recycling, and resource availability, such as when diadromous fish bring nutrients from the ocean to the terrestrial realms. But what is the broader role of altitudinal migration in such ecosystem services and how does this vary among different groups of altitudinal migrants? While habitat loss and broad‐scale global change have disrupted many ecosystems (Brodie et al., 2021; Wootton et al., 2023), how have changes in the migratory routes and abundances of altitudinal migrants affected ecosystem services? These declines and disruptions to migratory species also have impacts on the ecosystem services provided to humans such as food (i.e., dams disrupting fish migration Dugan et al., 2010) or money from ecotourism (i.e., Monarchs López‐Hoffman et al., 2017).

## 6 AN EXPANDING TOOLBOX FOR COMPARATIVE STUDIES OF ALTITUDINAL MIGRATION

Fortunately, for our ability to address these challenges, the toolbox to detect and study altitudinal migration is rapidly expanding and improving. Here, we describe key advancements and resources that the field can leverage toward a deeper understanding of the taxonomic prevalence and nature of altitudinal migration. Rather than restricting themselves to a single tool, future researchers will benefit from integrating these tools to answer when, where, how, and why animals move along elevational gradients across seasons (i.e., Ruegg et al., 2017).

1. Community science is steadily growing, adding thousands of observations that can be used to determine animal movements across seasons (Rueda‐Uribe et al., 2023; Tsai et al., 2021). Surveys and observational data provide a simple yet powerful way to detect changes in seasonal abundance across elevational gradients (Cheng et al., 2022; Liang et al., 2021). However, community science data also present unique challenges when applied to studies of altitudinal migration. Because many altitudinal migrants are partial migrants (Hsiung et al., 2018), sole reliance on presence and absence data may overlook some potential altitudinal migrants. Conversely, seasonal changes in abundance at an elevation may be the result of distant populations moving latitudinally rather than the local populations moving altitudinally (i.e., *Suiriri s. suiriri*, Chesser, 1997). Perceived changes in seasonal abundance at one elevational may be the result of seasonal change in a species behavior or detection bias (Werema, 2020) rather than migration. Using abundance data in combination with sex or age ratio data can provide information at the population level rather than describing the movement of individuals. Furthermore, effort and observers are not standardized, and this variation needs to be accounted for before assigning migratory status (Johnston et al., 2021, 2023; Strimas‐Mackey et al., 2023). Seasonal or regional differences in observational effort may lead to incorrect categorizations of migration.
2. Tracking technologies and remote sensing applications are improving at a remarkable pace: biologgers using satellite, radio, or acoustic transmitters are increasingly smaller, cheaper, and easier to use (Börger et al., 2020; Holton et al., 2021) while increase in coverage and improvements in remote sensing has given us an improved understanding in the ecology of migrants (i.e., Bastille‐Rousseau et al., 2017; Harvey & Larsen, 2020; Li et al., 2023). This revolution in tracking technologies has led to new discoveries in animal movement and migration (i.e., Satyr Tragopan *Tragopan satyra*, Norbu et al., 2013; *Patagona* hummingbirds, Williamson et al., 2024). New multi‐sensory tags that also record atmospheric pressure are especially well suited for short‐distance altitudinal migrants, and may help in our ability to distinguish diel‐ or weather‐related movements from seasonal migration across elevational gradients (Nussbaumer et al., 2023; Rhyne et al., 2024; Rime et al., 2023). Biologgers may also be used in studies beyond movement, but also at drivers, interactions, and learning in animal movement and migration (Beltran et al., 2024). Parasites and other symbionts offer another emerging framework to track populations as symbiont communities differ strongly across elevational gradients (Williamson & Witt, 2021).
3. Genomic data has long been used to study population connectivity among latitudinal migrants (e.g., DeSaix et al., 2019, 2023), but has not been as extensively applied to altitudinal migrants. Genomic data could be used to study gene flow among populations that differ in altitudinal migration behavior and could also be used to link populations between their breeding and non‐breeding distributions at different elevations, as has been done in many latitudinal migrants (e.g., Battey & Klicka, 2017). Further genomic data can also help our understanding of migratory connectivity of altitudinal migrants and if populations share breeding and non‐breeding sites across their annual cycle. Comparative and population genomics have identified various loci associated with altitudinal migration (i.e., Qu et al., 2015; Tigano & Russello, 2022), yet these studies have been relatively limited in scope and the degree to which altitudinal migration is an innate or learned behavior with a genetic component is unknown (Merlin & Liedvogel, 2019; Talla et al., 2020). Various studies have identified genomic loci associated with adaptations to hypoxic conditions at high‐elevation (Storz & Cheviron, 2021; Williamson et al., 2024), but our general understanding of the genetic underpinnings of altitudinal migration lags behind that of latitudinal migration (Justen & Delmore, 2022; Merlin & Liedvogel, 2019; Moussy et al., 2013; Rougemont et al., 2023; Sokolovskis et al., 2023; Toews et al., 2019).
4. Bulk stable isotope analysis—primarily of Hydrogen but also Oxygen—has been foundational in many recent studies that aim to detect altitudinal migration (Gadek et al., 2018; Newsome et al., 2015). In short, these studies approximate changes in location across time by quantifying associations between isotopic values of animal tissues and precipitation isotope values that vary across elevational gradients (Bowen, 2010). However, interpreting stable isotope data is sometimes difficult due to potentially confounding factors of shifting isotopic baselines and the influence of trophic cascades on isotope values (Hobson et al., 2012). The use of trace element isotopes and microchemistry has been suggested as a new technology to better detect altitudinal migration (Chapman et al., 2012; Hobson et al., 2019), yet has seen few applications to date in part due to high monetary costs and difficulty in obtaining and analyzing samples. The advent of compound‐specific stable isotope analyses of amino acids (CSIA‐AA) offers new possibilities and increased power to detect altitudinal migration by more directly connecting isotopes to the landscape rather than diet (McMahon & Newsome, 2019). For example, CSIA‐AA was used to trace the long‐distance migration of Chum Salmon (*Oncorhynchus keta*) between Okhotsk and Bering seas (Matsubayashi et al., 2020). In particular, CSIA‐AA of Hydrogen could provide improved spatial resolution for tracking altitudinal migrants compared to bulk stable isotope analyses (McMahon & Newsome, 2019). However, “isoscapes” that describe spatial patterns of compound‐specific isotopic variation are not yet available due to the specialized instrumentation and expenses required to process hundreds or thousands of samples at continental scales. As the technologies underlying isotopic analyses continue to improve, future studies of altitudinal migration incorporating CSIA‐AA will be better able to discriminate spatial from trophic signatures of isotopic values underlying altitudinal migration. Furthermore, isotopes can be used to detect changes in diet of altitudinal migrants during differing parts of the migration cycle as has been done in latitudinal migration (i.e., Carter et al., 2024).
5. Natural history collections offer spatial and temporal series of specimens that can be combined with aforementioned techniques to study how altitudinal migration may have changed over time during the Anthropocene (Schmitt et al., 2019). Many techniques used to estimate the geographic origin from contemporary samples can be applied to museum specimens, such as stable isotopes (Rocque & Winker, 2005) and historical DNA sequencing (Wandeler et al., 2007), providing a potential way to examine temporal shifts in altitudinal migration and improve our ability to detect and forecast future changes in migration. However, differences in preservation media—especially formalin—may impact stable isotope values (Edwards et al., 2022) and our ability to accurately sequence historical DNA (Do & Dobrovic, 2015). As natural history museums contribute specimens and metadata via continued collecting efforts and online databases (Nachman et al., 2023), additional studies of spatiotemporal change in altitudinal study will be unlocked.

## 7 CONCLUSION

Here, we have developed a taxonomically inclusive comparative framework for the study of altitudinal migration that considers the biological relevance of biotic and abiotic changes between distinct sites across the annual cycle. We suggest that migration phenomena should be compared with respect to the strength and nature of ecological and physiological changes imparted by seasonal movement along vertical and horizontal axes. A comparative framework for studying altitudinal migration acknowledges the complexities when classifying and comparing altitudinal migrants: many altitudinal migrants are partial migrants that also move across latitudinal gradients, and there is also a continuum between movement and migration that is sometimes difficult to partition. There is still considerable work to be done to characterize the taxonomic extent of altitudinal migration, understand regional differences in patterns of altitudinal migration among biomes, and mitigate anthropogenic impacts on altitudinal migrants. Armed with an expanding toolbox, researchers will benefit from a stronger comparative framework that enables discussion across a wider breadth of taxonomic groups, thereby revealing the evolutionary drivers, ecological interactions, and conservation risks of altitudinal migrants across aquatic and terrestrial biomes.

## AUTHOR CONTRIBUTIONS

**David Vander Pluym:** Conceptualization (equal); methodology (equal); writing – original draft (lead); writing – review and editing (equal). **Nicholas A. Mason:** Conceptualization (equal); methodology (equal); writing – original draft (supporting); writing – review and editing (equal).

## CONFLICT OF INTEREST STATEMENT

We report no conflict of interest.

## Supporting information


Data S1:

## Data Availability

The data from our literature search and the R code used to process those data are available via the Dryad Data Repository DOI: 10.5061/dryad.sn02v6xcv.
